# Effect of Breathing Oxygen-Enriched Air on Exercise Performance in Patients With Pulmonary Hypertension Due to Heart Failure With Preserved Ejection Fraction: A Randomized, Placebo-Controlled, Crossover Trial

**DOI:** 10.3389/fmed.2021.692029

**Published:** 2021-07-28

**Authors:** Julian Müller, Mona Lichtblau, Stéphanie Saxer, Luigi-Riccardo Calendo, Arcangelo F. Carta, Simon R. Schneider, Charlotte Berlier, Michael Furian, Konrad E. Bloch, Esther I. Schwarz, Silvia Ulrich

**Affiliations:** ^1^Pulmonary Clinic, University Hospital Zürich, Zürich, Switzerland; ^2^Faculty of Medicine, University of Zürich, Zürich, Switzerland; ^3^Centre for Integrative Human Physiology, University of Zürich, Zürich, Switzerland

**Keywords:** pulmonary hypertension, heart failure with preserved ejection fraction, oxygen therapy, exercise, external cycling work, cardiopulmonary exercise test

## Abstract

**Objective:** To evaluate the effects of breathing oxygen-enriched air (oxygen) on exercise performance in patients with pulmonary hypertension due to heart failure with preserved ejection fraction (PH-HFpEF).

**Methods:** Ten patients with PH-HFpEF (five women, age 60 ± 9 y, mPAP 37 ± 14 mmHg, PAWP 18 ± 2 mmHg, PVR 3 ± 3 WU, resting SpO_2_ 98 ± 2%) performed two-cycle incremental exercise tests (IET) and two constant-work-rate exercise test (CWRET) at 75% maximal work-rate (*W*_max_), each with ambient air (FiO_2_ 0.21) and oxygen (FiO_2_ 0.5) in a randomized, single-blinded, cross-over design. The main outcomes were the change in *W*_max_ (IET) and cycling time (CWRET) with oxygen vs. air. Blood gases at rest and end-exercise, dyspnea by Borg CR10 score at end-exercise; continuous SpO_2_, minute ventilation (V'E), carbon dioxide output (V'CO_2_), and cerebral and quadricep muscle tissue oxygenation (CTO and QMTO) were measured.

**Results:** With oxygen vs. air, *W*_max_ (IET) increased from 94 ± 36 to 99 ± 36 W, mean difference (95% CI) 5.4 (0.9–9.8) W, *p* = 0.025, and cycling time (CWRET) from 532 ± 203 to 680 ± 76 s, +148 (31.8–264) s, *p* = 0.018. At end-exercise with oxygen, Borg dyspnea score and V'E/V'CO_2_ were lower, whereas PaO_2_ and end-tidal PaCO_2_ were higher. Other parameters were unchanged.

**Conclusion:** Patients with PH-HFpEF not revealing resting hypoxemia significantly improved their exercise performance while breathing oxygen-enriched air along with less subjective dyspnea sensation, a better blood oxygenation, and an enhanced ventilatory efficiency. Future studies should investigate whether prolonged training with supplemental oxygen would increase the training effect and, potentially, daily activity for PH-HFpEF patients.

**Clinical Trial Registration:** [clinicaltrials.gov], identifier [NCT04157660].

## Introduction

In patients with heart failure with preserved ejection fraction (HFpEF), pulmonary hypertension (PH) is frequent and associated with reduced quality of life, exercise intolerance, disease progression, morbidity, and mortality (1, 2). Reduced exercise capacity in HFpEF has been associated with adverse outcome such as reduction in quality of life and increased mortality. Despite multiple international efforts, no pharmacological treatment has been identified that can effectively reduce mortality in HFpEF. However, with medical therapy a substantial improvement in exercise capacity without change in cardiac function has been reported in a meta-analysis by Holland et al. (3). Exercise training has been strongly associated with an increase in exercise capacity and quality of life in this patient collective, which is difficult to treat (4). Exercise intolerance in patients with heart failure has been attributed to multiple factors, including impaired cardiopulmonary reserve, a reduction of peripheral and respiratory skeletal muscle function, and mitochondrial dysfunction (1, 5, 6).

In healthy subjects, patients with pulmonary arterial (PAH) or chronic thromboembolic PH (CTEPH), or patients with chronic obstructive pulmonary disease (COPD), breathing oxygen-enriched air during cycling exercise is associated with an increased maximal work-rate (*W*_max_) and constant work-rate exercise test (CWRET) time compared to breathing ambient air (7–9). Several mechanisms of the oxygen pathway are improved with higher arterial oxygenation in those patients, namely, increased oxygen availability to the muscle and cerebral tissue and also improved ventilatory efficiency.

While long-term oxygen therapy is recommended for hypoxemic patients with PH-HFpEF (10), so far, no study has assessed the potential role of oxygen during exercise training in non-hypoxemic PH-HFpEF.

The purpose of the current randomized, sham-controlled trial is therefore to test the hypotheses that oxygen-enriched air (oxygen, FiO_2_ 0.50) increases exercise capacity in an incremental (ramp) exercise test (IET) and cycling time in a high-intensity CWRET in stable PH-HFpEF patients without resting hypoxemia, compared to ambient air (air, FiO_2_ 0.21).

## Materials and Methods

### Study Design

This randomized, placebo-controlled, single-blinded, cross-over trial evaluated the effect of oxygen therapy in improving exercise performance in non-hypoxemic patients with PH-HFpEF using IET and CWRET protocols twice, once with and once without oxygen supplementation in randomized order, as described previously (8, 9). The study was conducted in accordance with the amended declaration of Helsinki (11), approved by the institutional ethical committee (KEK 2012-0251), and registered at ClinicalTrials.gov (NCT04157660), and participants gave their written informed consent.

### Participants

Patients with PH-HFpEF diagnosed by right heart catheterization [pulmonary artery wedge pressure (PAWP) > 15 mmHg, mean pulmonary artery pressure (mPAP) > 25 mmHg] and normal left ventricular ejection fraction (LVEF > 50%), aged 18–80 years, were included (12). The participants were stable on the same medication for the last 4 weeks and had no relevant co-morbidities, no chronic lung disease, no musculoskeletal limitation, and were not pregnant. Any other unstable condition or contraindication for ergo-spirometry led to exclusion.

### Interventions

On two separate days, patients performed each two-cycle exercise tests at rates of 50–60 rpm to exhaustion: On the first day, patients underwent two IETs with increments of 10–20 W/min according to the patient's fitness; on the second day, two CWRETs at 75% of individual *W*_max_ achieved with air. In both tests (IET and CWRET), the work-rate started after 120 s of cycling without resistance. There was a recovery period for at least 2 h between the tests. For each test, patients were breathing either air or oxygen in a randomized order. Subjects were connected to the flow sensor of a metabolic unit via a mouthpiece (Ergostick, Geratherm Medical, Geschwenda, Germany) and a low-resistance two-way valve (Hans Rudolph, Shawnee, USA). The inlet of the valve was connected to a gas-mixing device to provide either air or oxygen (Altitrainer, SNTEC SA, Nyon, Switzerland). The metabolic unit was calibrated before each test. The patients were blinded to the oxygen settings.

### Assessments

Clinical and diagnostic assessments were performed as described previously (9, 13): At the screening visit, medical history, clinical examination, 6-min walk test (6MWT), and spirometry were noted. Prior to the test at interventional days, patients were not allowed to do sports and drink alcohol, coffee, or carbonated drinks and were instructed to take their regular medication and to eat a light meal more than an hour before.

Respiratory gas exchange was recorded with the metabolic unit. Breathing rate, tidal volume (Vt), minute ventilation (V'E), CO_2_ output (V'CO_2_), and derived variables were recorded breath by breath. Oxygen uptake is not reported for tests with oxygen because accuracy of the O_2_-sensor outside the calibration range of FiO_2_ 0.16–0.21 could not be verified. Heart rate was derived from a 12-lead ECG and blood pressure was measured by automated arm-cuff measurements. Finger pulse oximetry (SpO_2_) and cerebral and quadricep muscle tissue oxygenation (CTO, QMTO) were recorded by near-infrared-spectroscopy sensors (NIRO-200NX, Hamamatsu, Shizuoka, Japan) (14–16).

Physiological variables were recorded breath by breath and averaged over successive 30 s intervals. Variables at end-exercise were defined as a mean over the final 30 s before the termination of the exercise defined as a drop in cycling rate < 50 rpm. The ventilatory equivalents for V'CO_2_ were calculated as V'E/V'CO_2_ at end-exercise and V'E/V'CO_2_ as slope over the entire duration of ramp exercise (17). Isotime was defined as the final 30 s to end-exercise of the shorter test compared with the longer test at the same time point. Reference values for *W*_max_ were those reported by Glaser et al. (18).

### Main Outcome

Co-primary outcomes were the change in maximal work-rate during IET (part 1) and the change in cycling time during high-intensity CWRET (part 2).

### Randomization and Blinding

The two different conditions (air vs. oxygen) were randomized by software-based block randomization for the first test day. At the second test day the same order was maintained. Participants were blinded to the FiO_2_.

### Sample Size and Statistics

Based on previous studies on oxygen therapy in patients with pulmonary hypertension (8) and chronic obstructive pulmonary disease (9), a sample size estimation indicated that 10 participants were required to detect a difference of 5 W in IET and 2 min in CWRET (19, 20) with a power of 0.8 and alpha level of 0.05 for both trials. Normal distribution of data differences was assessed with the Kolmogorov–Smirnov test. Data are summarized as mean ± standard deviation (SD), mean difference, and 95% confidence interval (CI) between air and oxygen. As all participants had complete data sets, the results correspond to both an intention-to-treat and per-protocol analysis. Data from the tests (IET or CWRET) with air and oxygen were compared at end-exercise and at identical times corresponding to end-exercise with the shorter tests (isotime). Data from IET with air and oxygen were further compared over the progressive course of exercise at corresponding exercise times, respectively, work-rate at submaximal exercise intensity. We therefore computed the means over each 10% fraction of maximal exercise duration individually achieved with air, i.e., 1–10%, 11–20%, etc., up to 91–100% of the total exercise duration, and comparatively computed the corresponding means over identical time periods in tests with oxygen, as described before (9, 13). Linear mixed regression analyses were used to explore changes in *W*_max_ (IET) or cycling time (CWRET) induced by oxygen and adjusted for baseline variables [body mass index (BMI), age, sex, New York Heart Association Functional Classification (NYHA-Class), LVEF, 6-Min Walk Test (6MWT)]. To investigate the differences in delivered external work (J) with oxygen vs. air, the two tests (CWRET and IET) were compared by calculating the integral of power (W) over time (s). Significance was assumed at *p* < 0.05, with 95% CI not overlapping zero. Data were analyzed with the statistical programs SPSS Version 26 (SPSS, Chicago, IL, USA) and R studio version 1.2.1578, and the graphs were drawn with Matlab version R2017b.

## Results

**Figure 1:**
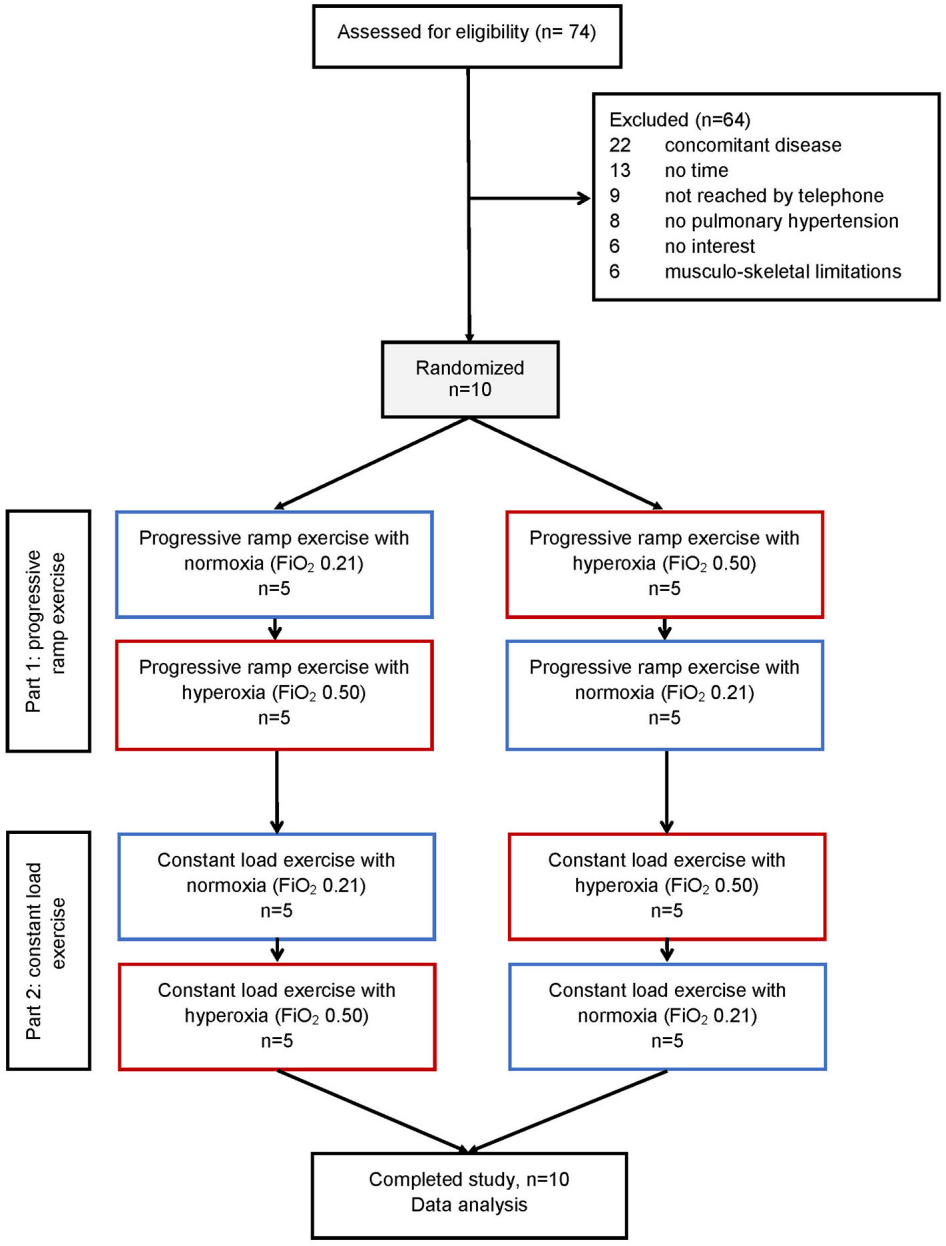
Patient flow of the study. *N* = Number of participants.

Figure 1 shows the patient flow, and baseline characteristics are presented in Table 1. Of seventy-four patients having been assessed for PH-HFpEF by right heart catheter, we were able to randomize 10 patients (two combined pre- and post-capillary, eight post-capillary PH) and these patients completed all tests according to the protocol between September 2019 and January 2020. The patients were on average 60 ± 9 years old with a BMI of 28 ± 6 kg/m^2^. Five of 10 were female, and all were on stable medical treatment for heart failure according to guidelines and revealed comorbidities, as expected. Mean LVEF was 63 ± 5%, SpO_2_ at rest 98 ± 2% and 6-min walking distance 495 ± 54 m (Table 1).

**Table 1:** Baseline characteristics.

Number of participants	10
Female; male	5; 5
Age (years)	60 ± 9
Body mass index (kg/m^2^)	28 ± 6
New York Heart Association functional class I/II	7 (70%)
New York Heart Association functional class III	3 (30%)
Left ventricular ejection fraction (%)	63 ± 5
**Medication:**	***N (%)***
Diuretics	10 (100)
beta-Blocker	3 (30)
ACE inhibitors	3 (30)
AT-2 blocker	1 (10)
Calcium antagonists	6 (60)
**Right heart catheterization data:**	
Heart rate (bpm)	74 ± 10
Systolic blood pressure (mmHg)	140 ± 25
Diastolic blood pressure (mmHg)	77 ± 8
Mean pulmonary artery pressure (mmHg)	37 ± 14
Pulmonary artery wedge pressure (mmHg)	18 ± 2
Cardiac index (L/min/m^2^)	3.4 ± 0.8
Pulmonary vascular resistance, (WU)	3 ± 3
Pulmonary vascular resistance >3WU	2
SpO_2_ rest (%)	98 ± 2
6-min walking distance (m)	495 ± 54
SpO_2_ at end of 6-min walk (%)	95 ± 5
**Co-morbidities:**	***N (%)***
Obesity	2 (20)
Arterial hypertension	3 (30)
Coronary heart disease	1 (10)
Diabetes	2 (20)
Cardiomyopathy	1 (10)
Pulmonary emphysema	1 (10)
Atrial fibrillation	1 (10)
Systematic sclerosis	1 (10)
Hypercholesterolemia	3 (30)

*Values are presented as mean ± SD or total number (%). ACE, angiotensin converting enzyme; AT-2, angiotensin-2 receptor; SpO_2_, arterial oxygen saturation*.

### Incremental (Ramp) Cycle Exercise

**Figure 2:**
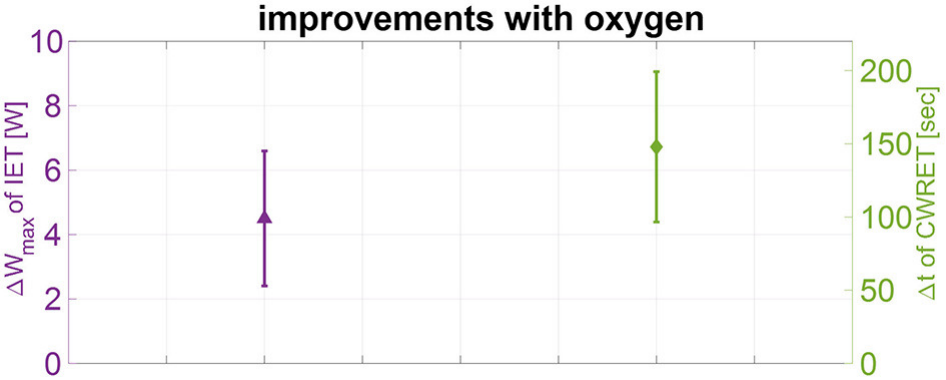
Improvements of the primary outcomes with oxygen. Changes of maximal work-rate [*W*_max_ (W)] at the incremental exercise test (left, purple) and mean changes of maximal cycling time [t (s)] of the constant work-rate exercise test (right, green) with oxygen-enriched vs. ambient air are shown as mean ± standard error of mean (SEM). IET, incremental exercise test; CWRET, constant work-rate exercise test.

**Figure 3:**
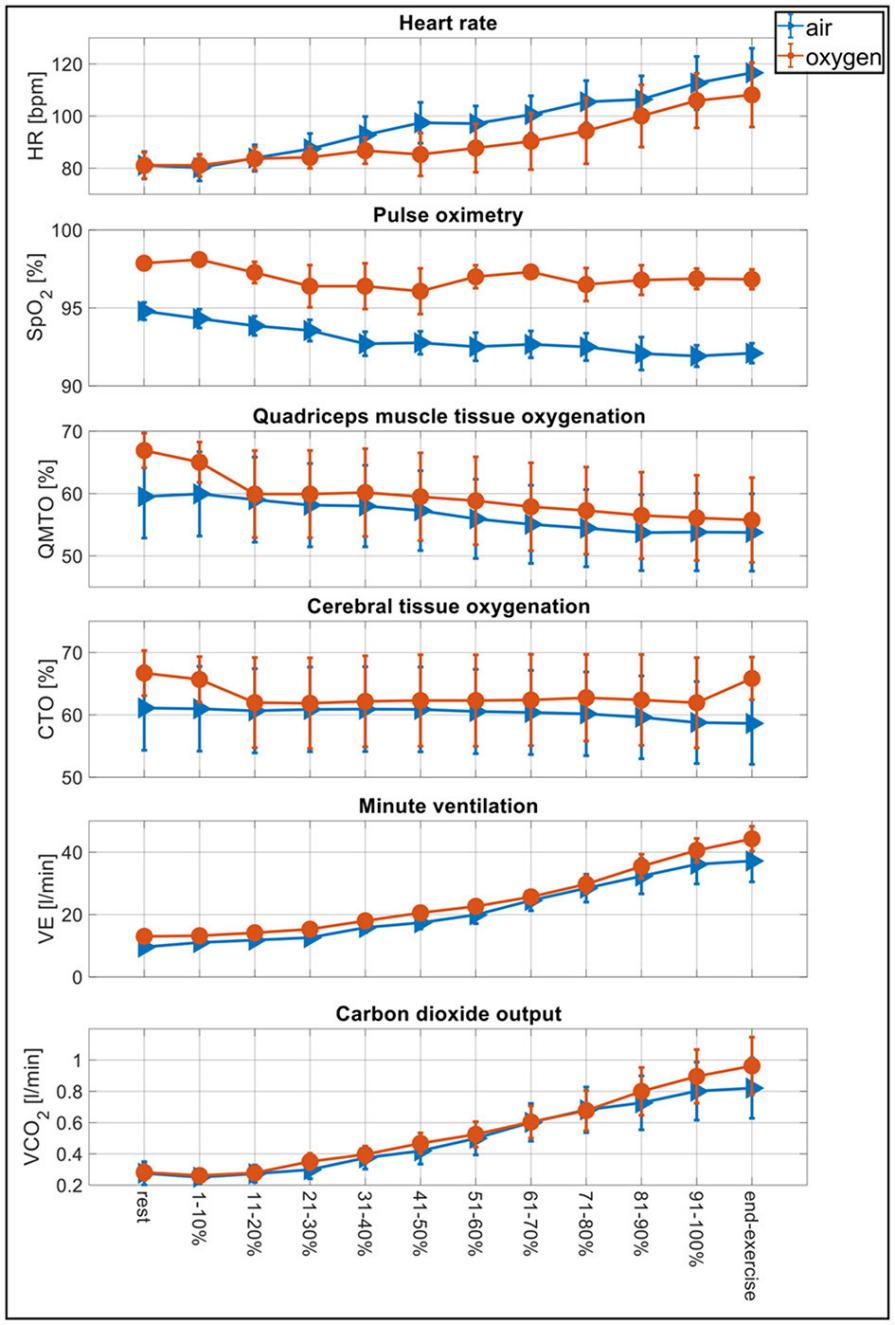
Physiological changes during the incremental exercise test. Physiological changes of the incremental exercise test comparing tests with air (blue) vs. oxygen-enriched air (red) by computing means over each 10% fraction of maximal exercise duration individually achieved with ambient air, i.e., 1–10%, 11–20%, etc., up to 91–100% of total exercise duration and end-exercise defined as the mean over the final 30 s of the test. HR, heart rate; SpO_2_, arterial oxygen saturation; QMTO, quadriceps muscle tissue oxygenation; CTO, cerebral tissue oxygenation; V'E, minute ventilation; V'CO_2_, carbon dioxide output.

Table 2 and Figures 2 and 3 summarize the results of the IET. With air, *W*_max_ was reduced compared to predicted normal values (94 ± 36 W, 61 ± 14% predicted). Oxygen significantly increased *W*_max_ to 99 ± 36.3 W, corresponding to a mean change (95% confidence interval) of +5.4 W (0.9–9.8, *p* = 0.025). At end-exercise, patient-reported Borg CR10 dyspnea score was significantly reduced with oxygen from 6.9 ± 1.7 to 6.1 ± 1.3, corresponding to a mean change of −0.8 (−1.5 to −0.1, *p* = 0.039). Borg CR10 scores for leg discomfort did not differ from values under air.

**Table 2:** Results of the incremental exercise test.

**IET**	**Air**	**Oxygen**			
	**End-exercise**	**End-exercise**		**Isotime**	
	**Mean**	**Mean**	**Difference (95% CI)**	**Mean**	**Difference (95% CI)**
Maximal work-rate (W)	93.6 ± 36.2	99 ± 36.3^*^	5.4 (0.9/9.8); +6% (1/11)	93 ± 36	NA
Exercise time (s)	481 ± 128	540 ± 133^**^	60 (33/86)	478 ± 122	NA
Heart rate (bpm)	117 ± 30	108 ± 39	−9 (−38/22)	108 ± 44	−18.1 (−52/16)
Heart rate reserve (bpm)	41 ± 32	45 ± 40	4 (−25/34)	37.4 ± 28.1	3 (−2/8)
V'E (L/min)	39.6 ± 22	44 ± 12	4.7 (−8/17)	43 ± 13	2 (−12/16)
Breathing reserve (%MVV)	61 ± 28	57 ± 25	−4 (−15.7/6.8)	57.8 ± 25.1	−2 (−14.3/10.3)
Tidal volume (l)	1.1 ± 0.6	1.2 ± 0.6	0.1 (−0.3/0.4)	1.16 ± 0.65	0.09 (−0.3/0.5)
Breathing rate (1/min)	30.4 ± 5.2	30.4 ± 6	0 (−2.6/2.4)	29.4 ± 6.3	−2.7 (−6.5/1.1)
V'CO_2_ (L/min)	0.8 ± 0.6	1.0 ± 0.6	0.2 (−0.1/0.4)	0.93 ± 0.57	0.09 (−0.2/0.4)
V'E/V'CO_2_	34.8 ± 3	31.7 ± 2.6^**^	−3.1 (−4.4/−1.8)	31.4 ± 2.8^**^	−3.8 (−5.4/−2.1)
V'E/V'CO_2_ slope	32.2 ± 5.1	29 ± 3.8^*^	−3.3 (−5.9/−0.6)	NA	NA
End-tidal PaCO_2_ (mmHg)	31.5 ± 5.4	34.7 ± 6.8^**^	3.2 (1.1/5.3)	35.1 ± 7.0^**^	4 (1.9/6.1)
Systolic blood pressure (mmHg)	157 ± 27	163 ± 20	6 (−8/21)	157 ± 22	−3 (−12/6)
Diastolic blood pressure (mmHg)	81 ± 26	87 ± 30	6 (−13/26)	90 ± 29	1.4 (−8/11)
SpO_2_ (%)	92 ± 2	97 ± 2^**^	5 (3/7)	97 ± 2^**^	5 (3/6)
CTO (%)	59 ± 21	66 ± 11	7 (−6/20)	62 ± 23	3 (−4/10)
QMTO (%)	54 ± 20	56 ± 22	2 (−2/6)	56 ± 22	2 (−3/7)
Arterial pH	7.39 ± 0.05	7.39 ± 0.05	0 (−0.03/0.02)	NA	NA
PaO_2_ (kPa)	11.7 ± 3.3	26.5 ± 10^**^	14.8 (5.9/23.6)	NA	NA
PaCO_2_ (kPa)	4.6 ± 0.5	4.8 ± 0.7	0.2 (−0.1/0.5)	NA	NA
SaO_2_ (%)	96 ± 3.6	99 ± 0.7^*^	3 (0.6/5.9)	NA	NA
Arterial lactate (mmol/L)	4.4 ± 2.7	4.6 ± 1.8	0.2 (−1.6/2.0)	NA	NA
Arterial HCO_3_ (mmol/L)	21.9 ± 2	22.3 ± 2.2	0.4 (−0.6/1.5)	NA	NA
Borg CR10 dysp. score	6.9 ± 1.7	6.1 ± 1.3	−0.8 (−1.5/−0.1)	NA	NA
Borg CR10 leg score	7.3 ± 1.3	7.4 ± 1.5	0.1 (−0.5/0.7)	NA	NA

*Values are presented as mean ± SD, n = 10. End-exercise represents the values averaged over the last 30 s. Isotime refers to the identical time of end-exercise of the shorter test. MVV, maximal voluntary ventilation; SpO_2_, pulse oximetry; CTO, cerebral tissue oxygenation; QMTO, quadriceps muscle tissue oxygenation; PaO_2_, arterial partial pressure of oxygen; PaCO_2_, arterial partial pressure of carbon dioxide; SaO_2_, arterial oxygen saturation; NA, not available*.

* *p < 0.05;*

** *p < 0.01*.

Oxygen increased SpO_2_, CTO, and QMTO by 5, 7 and 2% at end-exercise compared with air (Figure 3). Arterial blood gas analysis at maximal exercise revealed an increase in SaO_2_ and PaO_2_ under oxygen vs. air, while arterial pH remained unchanged even though the arterial lactate was slightly but not significantly higher under oxygen compared to air. Heart rate was reduced at end-exercise under oxygen. Breathing reserve was reduced by −4% with oxygen, while breathing rate was unchanged.

At end-exercise as well as at isotime, V'CO_2_ and V'E were unchanged with oxygen, while V'E/V'CO_2_ at end-exercise and the V'E/V'CO_2_ slope were significantly reduced, indicating increased ventilatory efficiency while breathing oxygen. Vt was higher and end-tidal PaCO_2_ increased significantly under oxygen to 34.7 ± 6.8 mmHg, corresponding to a mean change of +3.2 mmHg (1.1–5.3, *p* = 0.007).

Linear mixed regression analyses revealed no correlations between baseline characteristics and the improvement of *W*_max_ with oxygen.

### Constant Work-Rate Cycle Exercise

The results of the CWRET at end-exercise and isotime under oxygen vs. air are summarized in Table 3. CWRET with 75% of *W*_max_ determined under air corresponded to a mean work-rate of 70 ± 27 W. Breathing oxygen improved cycling time from a mean value of 532 ± 203 s under air to 680 ± 276 s, corresponding to a mean change of 148 s (32–264, *p* = 0.018) (Figure 2). Under oxygen, 5/10 patients increased their exercise time by >10% and 3/10 by >40%. Two patients decreased cycling time with oxygen by 122 and 55 s, respectively.

**Table 3:** Results of the constant work-rate exercise test.

**CWRET**	**Air**	**Oxygen**			
	**End-exercise**	**End-exercise**		**Isotime**	
	**Mean**	**Mean**	**Difference (95% CI)**	**Mean**	**Difference (95% CI)**
Cycling time (s)	532 ± 203	680 ± 276^*^	148 (32/264) +28% (6/50)	520 ± 200	NA
Work-rate (W)	70 ± 27	70 ± 27	NA	70 ± 27	NA
Heart rate (bpm)	121 ± 30	120 ± 29	−1 (−5/4)	117 ± 29^*^	−4.5 (−9/−1)
Heart rate reserve (bpm)	47 ± 49	34 ± 31	−13 (−42/16)	37 ± 32	−8.9 (−38/20)
V'E (L/min)	47 ± 12	37 ± 13	−9.6 (−20/1)	39 ± 8^**^	−9 (−10/−7)
Breathing reserve (%MVV)	62.7 ± 21.3	68.6 ± 16.3	5.9 (−2.5/14.3)	70 ± 15.6	9 (−0.6/20)
Tidal volume (L)	1.3 ± 0.7	1.2 ± 0.6	−0.1 (−0.4/0.1)	1.2 ± 0.6	−0.1 (−0.4/0.2)
Breathing rate (L/min)	31.5 ± 7.0	29.1 ± 5.4	−2.4 (5.3/0.5)	25.5 ± 3.9^*^	−5.9 (−10.7/−1.1)
V'CO_2_ (L/min)	0.9 ± 0.5	0.9 ± 0.5	−0.06 (−0.3/0.1)	0.9 ± 0.5	−0.1 (−0.4/0.2)
V'E/V'CO_2_	36.6 ± 4.6	32.2 ± 3.8^**^	−4.3 (−7.3/−1.5)	31.5 ± 2.9^**^	−5.8 (−8.3/−3.2)
End-tidal PaCO_2_ (mmHg)	31.1 ± 4.0	34.2 ± 4.4^**^	3.1 (1.1/5.1)	35.4 ± 4.3^**^	4.3 (2.6/6.1)
Systolic blood pressure (mmHg)	180 ± 34	160 ± 36	−20 (−43/4)	166 ± 38	−1 (−34/31)
Diastolic blood pressure (mmHg)	87 ± 24	81 ± 20	−6 (−30/18)	78 ± 22	7 (−33/19)
SpO_2_ (%)	91 ± 7	95 ± 4	4 (−1/9)	95 ± 4	4 (−1/8)
CTO (%)	57 ± 20	65 ± 12	8 (−10/26)	98 ± 3^*^	3 (1/6)
QMTO (%)	57 ± 21	58 ± 21	1 (−5/5)	64 ± 10	7 (−9/24)
Arterial pH	7.39 ± 0.06	7.38 ± 0.06^*^	−0.01 (−0.03/−0.0)	NA	NA
PaO_2_ (kPa)	9.4 ± 1.7	32.8 ± 4.6^**^	23.4 (19.2/27.6)	NA	NA
PaCO_2_ (kPa)	4.6 ± 0.4	4.8 ± 0.3	0.25 (−0.1/0.6)	NA	NA
SaO_2_ (%)	93.7 ± 4.1	99.8 ± 0.2^**^	6.1 (−9.9/−2.25)	NA	NA
Arterial lactate (mmol/L)	5.9 ± 2.9	5.2 ± 2.7	−0.7 (−1.9/0.6)	NA	NA
Arterial HCO_3_ (mmol/L)	21.9 ± 2.9	21.9 ± 3.0	0.04 (−1.2/1.1)	NA	NA
Borg CR10 dysp. score	7.4 ± 1.6	6.1 ± 2.0	−1.3 (−2.6/−0.04)	NA	NA
Borg CR10 leg score	6.8 ± 1.7	6 ± 1.6	0.8 (−0.8/2)	NA	NA

*Values are presented as mean ± SD, n = 10. End-exercise represents the values averaged over the last 30 s. Isotime refers to the identical time of end-exercise of the shorter test. MVV, maximal voluntary ventilation; SpO_2_; pulse oximetry; CTO; cerebral tissue oxygenation; QMTO; quadriceps muscle tissue oxygenation; PaO_2_, arterial partial pressure of oxygen; PaCO_2_, arterial partial pressure of carbon dioxide; SaO_2_, arterial oxygen saturation; NA, not available*.

* *p < 0.05;*

** *p < 0.01*.

At end-exercise breathing reserve increased by 6% under oxygen, while breathing rate was reduced. The patients perceived significantly less dyspnea under oxygen, decreasing Borg CR10 dyspnea scores from 7.4 ± 1.6 to 6.1 ± 2.0, corresponding to a mean change of −1.3 (−2.6 to −0.1, *p* = 0.045). SpO_2_ and CTO both increased by 4.0 and 7.9% under oxygen, whereas QMTO was unchanged. Arterial blood gas analysis resulted in higher PaO_2_, SaO_2_, and PaCO_2_ with oxygen than air but lower lactate concentrations.

At isotime, heart rate under oxygen was significantly reduced corresponding to a mean change of −5 bpm (−8 to −1, *p* = 0.031), breathing reserve was increased by 9%, while breathing rate and V'E were significantly reduced. CTO, SpO_2_, and QMTO increased to 3, 4, and 7% with oxygen.

Linear mixed regression analyses revealed no correlations between baseline characteristics and improvement of cycling time with oxygen.

### External Cycling Work Delivery

**Figure 4:**
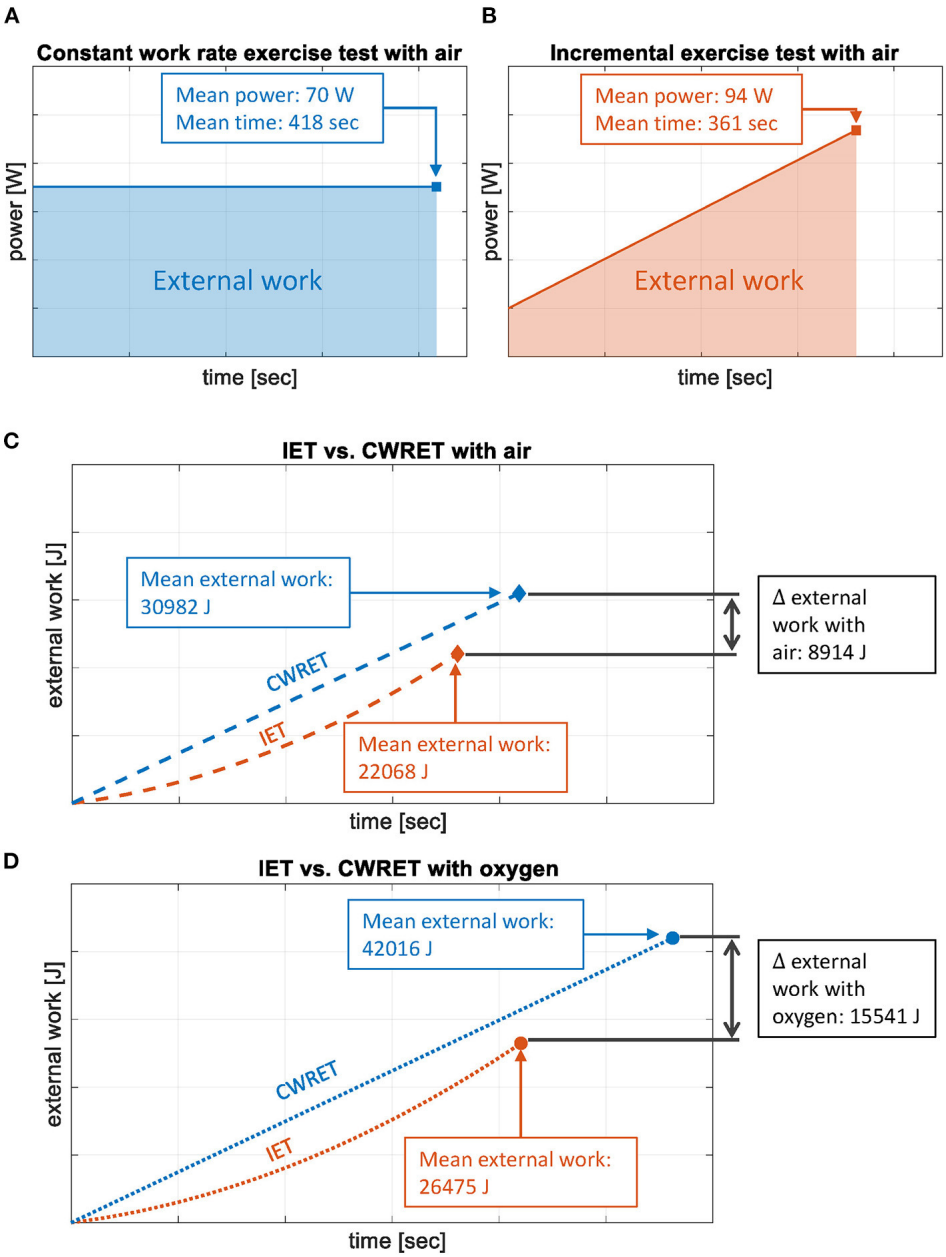
Cycling energy delivery during incremental vs. constant work-rate exercise test (IET vs. CWRET). **(A)** Graphical representation of the constant work-rate exercise test (CWRET, blue) with air for a mean cycling time of 418 s and a mean work-rate of 70W. **(B)** Graphical representation of the incremental exercise test (IET, red) with air for a mean cycling time of 361 s and a mean work-rate of 94W, starting with 20W. **(C)** Graphical representation of the mean delivered external work of the patients, calculated with the integral of the work-rate over cycling time, comparing both tests [CWRET (blue); IET (red)] with air. The curves represent the estimated progress with time for both tests. **(D)** Graphical representation of the mean delivered external work of the patients, calculated with the integral of the work-rate over cycling time, comparing both tests [CWRET (blue); IET (red)] with oxygen. The curves represent the estimated progress with time for both tests. ∫ W dt = external work (J).

In CWRET under air, participants cycled for a mean time of 418 s (under resistance, 538–120 s) with a mean work-rate of 70 W. In IET under air, participants cycled for a mean time of 361 s (under resistance, 481–120 s) with a mean work-rate of 94 W (Figures 4A,B). By integral calculation of work-rate over cycling time, the delivered mean external work with air results in 30,982 J during the CWRET and 22,068 J during the IET (Figure 4C). Under oxygen, patients cycled for a mean time of 560 s (680–120 s) with a mean work-rate of 70 W and delivered an external work of 42,016 J during CWRET and, for 420 s (540–120 s) with 99 W, they delivered 26,475 J of external work during IET (Figure 4D).

Accordingly, patients delivered significantly more external cycling work during the CWRET compared to the IET [+40% with air, mean change 8,914 J (1,522 to 16,306), *p* = 0.023; +59% with oxygen, mean change 15,541 J (2,953 to 28,129), *p* = 0.021].

Breathing oxygen was associated with a higher production of external work (CWRET: +36% and IET: +20%, *p* = 0.051 and 0.002).

## Discussion

This randomized, placebo-controlled, single-blinded, cross-over study in patients with PH-HFpEF without resting hypoxemia shows for the first time that breathing oxygen significantly enhances cycling exercise performance in terms of *W*_max_ in IET and cycling time in CWRET along with less dyspnea perception and a higher ventilatory efficacy.

The highest improvement of exercise performance was a 28% increase in CWRET cycling time (2.5 min) with oxygen compared to air, which is well above a postulated clinical important difference of 1.75 min for patients with COPD (19). This surpasses the relatively small improvement of 6% increase in *W*_max_ in the IET with oxygen. We assume that this is due to the fact that participants could exercise longer with the aerobic energy generating pathway in the CWRET, below the limit of the anaerobic threshold, which was in turn substantially raised by breathing oxygen.

The improvements in exercise performance with oxygen vs. air were associated with a higher blood oxygenation (SpO_2_, SaO_2_, PaO_2_) over the entire course of exercise (Figure 3). Similarly, albeit not significant, CTO and QMTO were improved, pointing toward a higher tissue oxygenation related to the higher arterial oxygen content and possibly an increased V'O_2_ with oxygen. By increasing F_i_O_2_ (normobaric hyperoxia) larger amounts of O_2_ will physically be dissolved in the arterial blood plasma and also bound to the hemoglobin to improve the SaO_2_ and increase the arterial oxygen content (21). It is supposed that breathing oxygen-enriched air (F_i_O_2_ > 0.21) triggers different cellular, molecular, neuronal, hormonal, and enzymatic responses that are leading to an ergogenic effect in maximal as well as submaximal loads (21–25). There is evidence that the intracellular pO_2_ could lead to more diffusion of O_2_ from the capillaries to the muscle cells (25); also there could be more effective oxygen storages with reduced dependencies to substrate phosphorylation with reduced anaerobic metabolism and different recruitment patterns of muscle fibers (26) to enhance exercise performance. In addition, the influence of the central nerve system by reducing fatigue as a “central governor” (22) or regulating hormonal release especially reduced levels of catecholamines (27, 28). Additionally, enzymatic protecting reactions like extracellular superoxide dismutase activity to reduce oxidative stress and damage (23) could all contribute essentially to the ergogenic effect of breathing oxygen-enriched air.

Therefore, exercise performance under oxygen may have been enhanced by improved tissue oxygenation by promoting the availability of oxygen in working muscles and in the cerebral motor and sensory neurons while reducing dyspnea perception despite the higher intensity and longer duration of exercise. In the literature, there is a growing body of evidence that suggests that non-cardiac factors like morphological changes in the periphery, such as skeletal muscles, may play an important role in exercise intolerance in HFpEF-patients. It is suspected that changes in mitochondrial structures, a decrease of capillary density, and a conversion of type 1 muscle fibers to type 2 muscle fibers may be the primary factors for impaired oxygen uptake and utilization (29, 30). The QMTO values measured in this study, which only increased marginally with oxygen, compared with other patient groups previously investigated, could support this theory (7–9).

Under oxygen, the maximum heart rate was reduced, an important finding in PH due to HFpEF patients, as a reduced heart rate signifies less autonomic stress, allows for increased cardiac filling, and is associated with a better prognosis and survival in heart failure (31). With the heart rate lowering effect of oxygen during physical exercise, patients reach their upper heart rate limit later and can therefore achieve higher exercise performance while breathing oxygen-enriched air.

Breathing oxygen was associated with a reduction of V'E/V'CO_2_ at end-exercise and the V'E/V'CO_2_ slope, while breathing rates and ventilation remained unchanged or reduced despite a longer exercise duration and/or higher work-rates, thus indicating an improved ventilatory efficiency. Furthermore, it has been shown that in patients with chronic heart failure, lower breathing rates increase arterial baroreflex sensitivity and improve exercise capacity (32).

Above the anaerobic threshold, the respiratory chain is omitted, and pyruvate is directly converted to lactate so the content of oxygen in the blood plays a subordinate role and the exercise performance depends mainly on the anaerobic capacity, as well as on the muscular leg strength. In all tests at end-exercise, lactate values were >4 mmol/L indicating a high acidic load just before cessation. However, arterial pH and bicarbonate concentration and breathing rate were unchanged, possibly indicating that maximal muscular exhaustion was reached before the maximal cardiorespiratory limitation.

Our previous trials in patients with precapillary PH and COPD demonstrated even larger exercise enhancing effects with oxygen compared to the presently investigated PH-HFpEF patients (8, 9), which might indicate that the beneficial effect of oxygen may be enhanced when exercise-induced or resting hypoxemia is present, like in patients with lung disease or PAH and CTEPH. However, other studies in healthy volunteers and athletes revealed that breathing oxygen-enriched air during exercise may be associated with an increase of up to 30% in maximal power output and up to 130% endurance time compared with ambient air, which indicates that the beneficial effects of oxygen therapy on exercise performance are consistently found in many different populations albeit to a varying magnitude (25, 33–37).

### Effect of Oxygen on Delivered External Cycling Work

Patients delivered significantly larger amounts of external work in the CWRET compared with the IET under both conditions (oxygen and air) despite the higher work-rate reached in shorter time in the IET. This may indicate that the patients did not stop the IET because of the exhausted energy resources but because of a lack of anaerobic capacity and leg muscle strength. For both tests (IET and CWRET), an increase of total delivered external work while breathing oxygen compared to air was found, even though the increase in maximal cycling time in CWRET was 28% with oxygen, whereas *W*_max_ in IET only increased by 6%. Despite higher relative increase in cycling time in CWRET compared to the *W*_max_ in IET, the increase in external work delivery was 36% in CWRET and 20% in IET with oxygen vs. air, indicating an improvement not only of endurance but also of the capacity of expending external cycling work and possibly enhanced work efficiency with oxygen breathing during exercise.

## Conclusion

This randomized, placebo-controlled trial showed for the first time a significantly improved exercise performance by breathing oxygen in patients with PH due to HFpEF in both IET and CWRET protocols, along with less dyspnea perception, better blood oxygenation, a lower maximal heart rate, and improved ventilatory efficiency. Comparison of IET and CWRET in terms of produced energy suggests that PH-HFpEF patients benefit from oxygen in both protocols but are able to produce higher energy levels on an aerobic energy-generating pathway. These improvements of exercise performance are of potential relevance to improve the training intensity and, potentially, the daily activity of PH-HFpEF patients.

## Data Availability Statement

The raw data supporting the conclusions of this article will be made available by the authors, without undue reservation.

## Ethics Statement

The studies involving human participants were reviewed and approved by Kantonale Ethikkomission Zürich. The patients/participants provided their written informed consent to participate in this study.

## Author Contributions

SU, ML, SS, ES, and KB contributed to the conception and design. JM, ML, SS, L-RC, AC, KB, ES, and SU contributed to the acquisition, analysis, or interpretation of data. JM, ML, SS, and SU drafted the manuscript. All authors revised the manuscript critically for important intellectual content.

## Conflict of Interest

The authors declare that the research was conducted in the absence of any commercial or financial relationships that could be construed as a potential conflict of interest.

## Publisher's Note

All claims expressed in this article are solely those of the authors and do not necessarily represent those of their affiliated organizations, or those of the publisher, the editors and the reviewers. Any product that may be evaluated in this article, or claim that may be made by its manufacturer, is not guaranteed or endorsed by the publisher.
